# Effects of different physical activities on brain-derived neurotrophic factor: A systematic review and bayesian network meta-analysis

**DOI:** 10.3389/fnagi.2022.981002

**Published:** 2022-08-26

**Authors:** Bojun Zhou, Zhisheng Wang, Lianghao Zhu, Gang Huang, Bing Li, Chaofan Chen, Junda Huang, Fuhai Ma, Timon Chengyi Liu

**Affiliations:** ^1^School of Physical Education and Sports Science, South China Normal University, Guangzhou, China; ^2^Academy of Plateau Science and Sustainability, Qinghai Normal University, Xining, China; ^3^School of Physical Education, Hubei Business College, Wuhan, China; ^4^School of Physical Education, Hunan University of Science and Technology, Xiangtan, China; ^5^Graduate School, Guangzhou Sport University, Guangzhou, China; ^6^School of Physical Education, College of Art and Physical Education, Gangneung-Wonju National University, Gangneung, South Korea; ^7^School of Physical Education, Xianyang Normal University, Xianyang, China; ^8^Qinghai Institute of Sports Science Limited Company, Xining, China

**Keywords:** exercise intervention, network meta-analysis, brain-derived neurotrophic factor, brain health, rehabilitation, quantitative difference, geometric mean

## Abstract

**Background:**

Emerging evidence suggests that exercise is a simple and effective method for maintaining brain function.

**Aims:**

This review evaluates the effects of five physical exercises, including aerobic training (AT), high-intensity interval training (HIIT), combined training (CT), resistance training (RT), and AT+RT, on the serum level of brain-derived neurotrophic factor (BDNF) in healthy and non-healthy populations.

**Methods:**

We searched CNKI, PubMed, Embase, Scopus, Medline, Web of Science, and Cochrane Library databases to review randomized controlled studies on exercise interventions for BDNF. Quantitative merging analysis of the resulting data using Bayesian network meta-analysis.

**Results:**

The screening and exclusion of the searched literature resulted in the inclusion of 39 randomized controlled trials containing 5 exercise interventions with a total of 2031 subjects. The AT, RT, AT+RT, HIIT, and CT groups (intervention groups) and the CG group (conventional control group) were assigned to 451, 236, 102, 84, 293, and 865 subjects, respectively. The Bayesian network meta-analysis ranked the effect of exercise on BDNF level improvement in healthy and non-healthy subjects as follows: RT > HIIT > CT > AT+RT > AT > CG. Better outcomes were observed in all five intervention groups than in the CG group, with RT having the most significant effect [MD = 3.11 (0.33, 5.76), *p* < 0.05].

**Conclusions:**

RT at moderate intensity is recommended for children and older adults in the case of exercise tolerance and is effective in maintaining or modulating BDNF levels for promoting brain health.

**Systematic Review Registration:**

https://inplasy.com, INPLASY202250164.

## Introduction

The central nervous system (CNS) is the primary site of distribution for brain-derived neurotrophic factor (BDNF), the second neurotrophic factor identified after nerve growth factor (NGF) (Binder and Scharfman, 2004). BDNF is most abundant in hippocampal and cortical tissues, and its ability to keep brain cells active promotes the survival, differentiation, and growth of brain neurons (Hans, 1995; Thoenen, 1995; Min-Wook et al., 2005). BDNF not only maintains and promotes neuronal development and differentiation along with nerve growth and regeneration but also engages in a variety of mechanisms such as regenerative repair after neuronal injury and neuronal degeneration prevention. In children and adolescents, BDNF is critical in supporting brain development and plasticity (Iughetti et al., 2011; Wrigglesworth et al., 2019). Physical activity increases the release of BDNF, insulin-like growth factor-1 (IGF-1), fibroblast growth factor 2 (FGF-2), NGF, and vascular endothelial growth factor (VEGF) (Gómez-Pinilla et al., 1997; Fabel et al., 2003; Griffin et al., 2011; Hong et al., 2015; Stein et al., 2018). These promote cerebral angiogenesis and penetrate the blood-brain barrier (BBB) and the blood-cerebrospinal fluid (CSF) barrier, affecting brain health; in the elderly population, reduced levels of BDNF and its receptors may be a major cause of the onset and progression of neurodegenerative diseases (Blesch, 2006; Ruiz-González et al., 2021), resulting in impaired brain health, memory loss, and cognitive decline in the absence of timely and effective interventions. In addition, BDNF is closely associated with cognitive function, whereas physical activity increases BDNF mRNA expression in the hippocampus, which remains high after a short period of cessation of exercise (Neeper et al., 1996; Shawne et al., 1996; Carl and Berchtold, 2002). As a result, changes in BDNF concentrations in the body may be directly linked to the mechanisms through which physical activity reshapes the brain, boosts cognition, and improves neurological disorders (Flöel et al., 2010; Knaepen et al., 2010; Erickson et al., 2011; Nascimento et al., 2015).

Exercise is frequently used in complementary and alternative medicine as a non-pharmacological treatment with hardly any side effects. Exercise therapy has been used extensively in recent years to intervene in nervous system diseases (NSDs), such as cognitive impairment, multiple sclerosis, and Parkinson's disease. There is research evidence indicating that exercise can increase serum BDNF levels, and BDNF is a protective factor for depression and NSDs (Guo et al., 2020). Some studies have shown that aerobic training (AT), resistance training (RT), high-intensity interval training (HIIT), and combined training (CT) do not, however, increase serum BDNF levels (Goekint et al., 2010; Krogh et al., 2014; Nicolini et al., 2019; Abbaspoor et al., 2020). BDNF levels are an important indicator of human brain health, and the overall findings on the effects of exercise therapy on BDNF levels in healthy subjects and patients with NSDs are worthy of further investigation for application in clinical practice. This study collected past RCTs and analyzed the effects of five different exercise interventions on serum BDNF levels in healthy and non-healthy populations using the Bayesian NMA method, yielding comprehensive comparative results (Lumley, 2002). Compared with traditional Meta-analysis, Network meta-analysis (NMA) allows the quantitative comparisons of different interventions for similar health problems to be pooled to integrate direct and indirect comparisons and produce better mixed estimates; provides advantages and disadvantages of the results of different interventions and ranks them, providing an “action guide” for the development of exercise prescriptions. The objective of our study was to understand the effectiveness of exercise in maintaining and promoting brain health in patients with degenerative neurological diseases vs. healthy populations and to provide more comprehensive and effective non-pharmacological evidence for subsequent clinical decisions.

## Materials and methods

The preferred reporting items for systematic reviews and meta-analyses (PRISMA) were used to conduct the NMA (Hutton et al., 2016). This study was registered with the International Platform of Registered Systematic Review and Meta-analysis Protocols (INLASY) under the unique identification number INPLASY202250164.

### Data sources and search strategy

CNKI, PubMed, Embase, Scopus, Medline, Web of Science, and the Cochrane Library were among the databases searched. In addition, ongoing and completed trials published between January 1990 and May 2022 were obtained from the American Clinical Trial Registry and the Chinese Clinical Trial Register. A combination of subject terms and free terms were used in the search for comprehensiveness and accuracy, and they were linked using Boolean logic operators. Supplementary material 1 provides a detailed search strategy using the PubMed database as an example.

### Literature inclusion and exclusion criteria

The inclusion criteria for the study were determined according to the PICOS criteria (Liberati et al., 2009). The detailed inclusion/exclusion criteria are reported in Tables 1, 2.

**Table 1:** Selection criteria.

**Category**	**Inclusion criteria**	**Exclusion criteria**
Population	All healthy and non-healthy people, irrespective of sex.	Incomplete data or data literature not available after email contact to the authors
Interventions	Supervised or unsupervised exercise interventions such as AT, RT, AT+RT, HIIT, CT, and other exercise therapies.	Lack of primary outcome indicators; non-English and non-Chinese literature
Comparison	The control group was no exercise intervention or a different modality of exercise than the observation group	Only one study with complete data was retained for duplicate publications or studies with highly consistent overall data
Outcomes	BDNF variation level	Exercise intervention duration ≤ 2 weeks
Study design	RCTs with two or more arms	Animal trials and non-RCTs

**Table 2:** Definition of exercise intervention.

**Intervention measures**	**Explanations**
AT	This is the type of exercise where the blood carries enough oxygen to the working muscles and is often characterized by low intensity and prolonged activity (Mersy, 1991).
RT	Applied using various types of equipment (e.g., free weights, elastic bands/tubing, weight machines), or simply by using the weight of a body segment or segments against gravity to provide resistance to training methods, for improving muscle strength or muscle endurance (Busch et al., 2013).
AT+RT	The total duration of the exercise intervention remained the same, with strength training and aerobic training performed separately to improve cardiorespiratory endurance and muscle strength/endurance.
HIIT	Bursts of exercise are performed in short periods of time, maximize exercise intensity (Gibala et al., 2012).
CT	Also known as multimodal training, refers to combined strength, flexibility, balance, and endurance exercises included in one program (Chaabene et al., 2021).

### Literature screening and data extraction

Two researchers (BJ-Z and ZS-W) read the titles and abstracts to initially screen the literature and reviewed the remaining literature to select studies that met the inclusion criteria. Subsequently, the screening results of the two researchers were exchanged and compared to confirm whether their results were consistent. Disagreements were discussed and addressed by the research team. Corresponding authors were contacted by email for data not published in the literature. In addition, data presented in graph form were extracted using GetDate software. The extracted information comprised basic information about the included studies (first author and year of publication), subject characteristics (age and sample size), and information on the exercise interventions (means of intervention for the test and control groups, number of interventions, intervention duration, and conclusions).

### Methodological assessment

Two researchers (BJ-Z and ZS-W) independently evaluated the methodology of a single RCT using the Cochrane Collaboration tool (Cumpston et al., 2019). They were evaluated based on random sequence generation, allocation concealment, participant personnel blinding, outcome assessment blinding, incomplete outcome data, selection bias, and other biases.

### Statistical methods

The effects of the five interventions were statistically analyzed using the R-Studio 4.1 and Addis 1.16.5 softwares, and the network diagram and sequence diagram of various interventions were plotted. NMA was started by R language programming, and Bayesian Markov Chain Monte Carlo (MCMC) algorithm was invoked by relevant instructions to analyze and map the results of random-effects model data (van Valkenhoef et al., 2013; Lin et al., 2014). The iterative convergence was evaluated using the potential scaling reduction factor (PSRF), and the statistical requirement was met when PSRF approached 1 (Van Valkenhoef et al., 2012). The results of the random-effects model data were previously evaluated and processed by the Bayesian MCMC algorithm invoked by relevant ADDIS statistical software instructions; *P* < 0.05 and 95% confidence intervals (95% CI) were used as the criteria for statistical difference. Due to the inclusion of less than 7 items of resistance training, and some studies did not report single load intensities and durations (Fragala et al., 2014; Deus et al., 2021), therefore no dose effect analysis was performed.

## Results

### Literature search results

**Figure 1:**
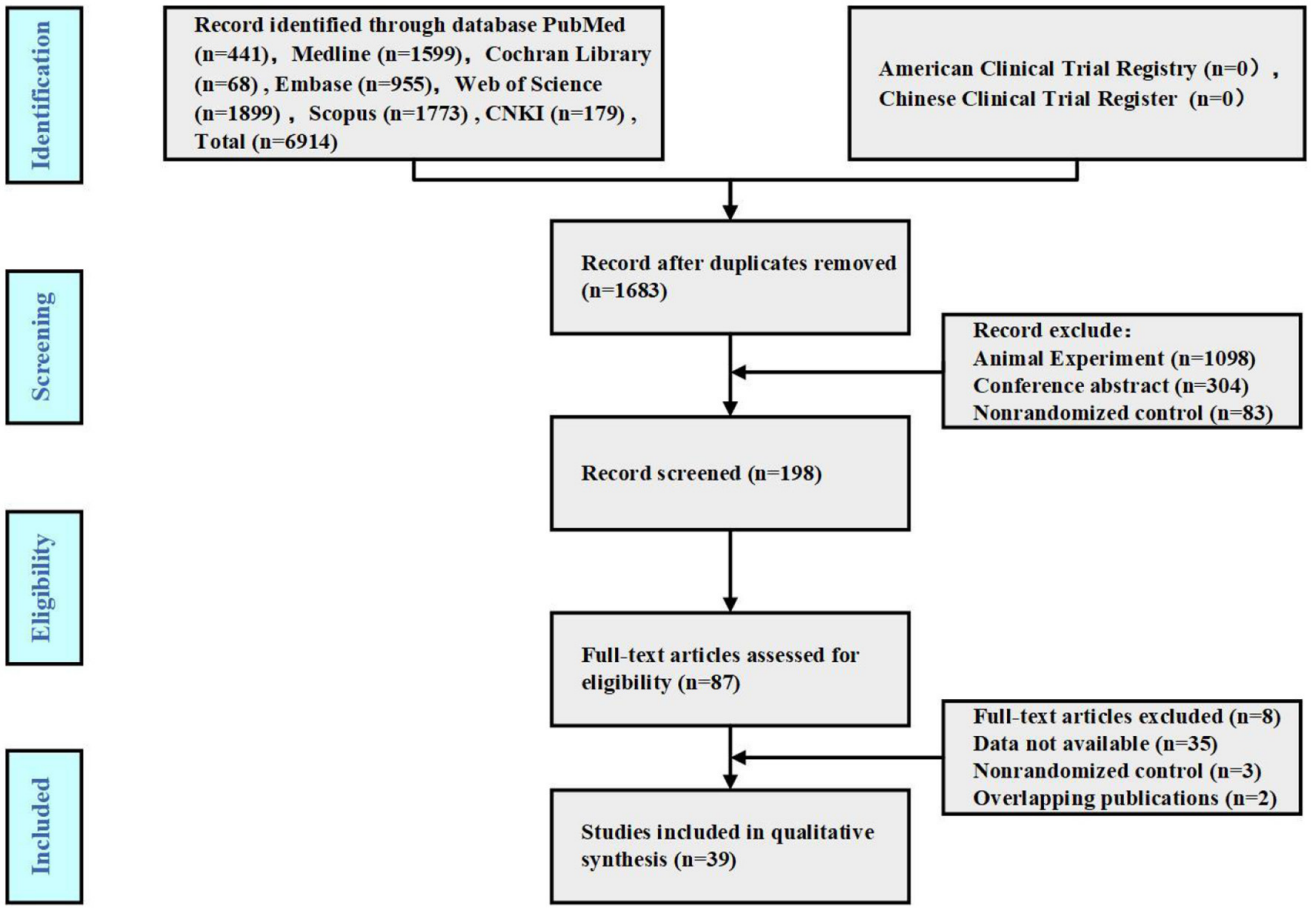
Study flow diagram.

According to the search strategy, 6,914 publications were initially obtained. After deleting duplicate studies using EndnoteX8, 39 publications were included. The specific search process is shown in Figure 1.

### Basic characteristics of the included literature

This study comprised 39 RCTs with a total of 2031 subjects ranging in age from 15.60 to 92.3 years. The AT, RT, AT+RT, HIIT, and CT groups (intervention groups) and CG group (conventional control group) were assigned to 451, 236, 102, 84, 293, and 865 subjects, respectively. The primary interventions were (1) AT, which included treadmill training, cycling, and aerobic gymnastics; (2) RT, which included large muscle group training, leg press, leg curl, leg extension, vertical traction, arm curl, and chest press; (3) AT+RT; (4) HIIT; and (5) CT, integrating RT, AT, balance training, and agility training. There was a wide variation in the interventions included in the study. The duration of interventions ranged from 15 to 75 min, with the majority lasting between 30 and 60 min. In addition, the frequency of training ranged from 2 to 7 times per week, with the minimum and maximum intervention durations of 3 weeks and 6 months, respectively. In addition, forearm venous blood samples were collected from each subject before and after the exercise intervention, and finally, BDNF concentrations were determined using enzyme-linked immunosorbent assay (ELISA); 36 studies examined serum samples from subjects and 3 studies examined plasma samples from subjects (Seifert et al., 2010; Vaughan et al., 2014; Nascimento et al., 2015). The detailed inclusion characteristics are shown in Table 3.

**Table 3:** Characteristics of the included studies.

**References**	**Sample size**	**Age(year)**	**Health** **Status**	**Session** **time**	**Frequency**	**Intensity**	**Interventions**	**Intervention duration**	**Control**	**Changes in BDNF concentration**
Vaughan et al. (2014)	CG = 23	CG = 68.8 ± 3.5	Health	60 min	Weekly/2 times	NA	Cardiorespiratory fitness, RT, and coordination and agility training, with instructor supervision and guidance during the intervention period.	16 weeks	Regular activities	CT_pre_ = 4.5
	CT = 25	CT = 69 ± 3.1								CT_post_ = 5.2
Arrieta et al. (2020)	CG = 45	CG = 84.70 ± 6.1	Health	30 min	Weekly/7 times	40–70% 1-RM	Exercises including strength, balance, and walking recommendations.	6 months	Routine activities	CT_pre_ = 34.2 (ng/ml)
	CT = 43	CT = 85.10 ± 7.6								CT_post_ = 33.5 (ng/ml)
Goldfield et al. (2018)	CG = 69	CG = 15.60 ± 1.3	Health	20–45 min	Weekly/4 times	AT: 65–85% HR_max_;	AT: exercising on a treadmill or indoor bicycle.	22 weeks	Diet control	AT_pre_ = 24.6 (ng/ml)
	AT = 69	AT = 15.50 ± 1.3				RT: moderate intensity to 8 times (8-RM)	RT: seven exercises using weight machines or free weights.			AT_post_ = 26.4 (ng/ml)
	RT = 70	RT = 15.80 ± 1.5				AT+RT: NA	AT+RT: complete AT and RT in each session.			RT_pre_ = 29.7 (ng/ml)
	AT+RT = 74	AT+RT = 15.5 ± 1.3								RT_post_ = 27.7 (ng/ml)
										AT+RT_pre_ = 27.9 (ng/ml)
										AT+RT_post_ = 26.2 (ng/ml)
Ledreux et al. (2019)	CG = 39	NA	Health	35 min	Weekly/5 times	NA	18 aerobic exercise routines.	5 weeks	Cognitive training	AT_pre_ = 24.5 (ng/ml)
	AT = 29									AT_post_ = 24.8 (ng/ml)
Ghafori et al. (2018)	CG = 20	CG = 10.2 ± 3.4	Health	30–45 min	Weekly/3 times	NA	Perform 3 different types of exercise,included exercises comprising fine, gross and motor exercises.	12 weeks	Regular activities	CT_pre_ = 542.47 (pg/ml)
	CT = 20	CT = 10.4 ± 3.5								CT_post_ = 642.80 (pg/ml)
Heisz et al. (2017)	CG = 32	CG = 20.5 ± 2.8	Health	20 min	Weekly/3 times	90–95% peak HR	20 min of high-intensity interval training	6weeks	Sedentary control	HIIT_pre_ = 33.5 (ng/ml)
	HIIT = 34	HIIT = 20 ± 2.7								HIIT_post_ = 31.3 (ng/ml)
Wagner et al. (2015)	CG = 17	CG = 23.7 ± 1.7	Health	60 min	Weekly/3 times	68–86% VO_2max_	Indoor cycling training	6 weeks	Regular exercise	AT_pre_ = 12.4 (ng/ml)
	AT = 17	AT = 25 ± 3.3								AT_post_ = 16.7 (ng/ml)
Cho and Roh (2016)	CG = 8	CG = 22.3 ± 2.1	Health	40 min	Weekly/3 times	70% HRR	NA	8 weeks	Dietary counseling	AT_pre_ = 24.8 (ng/ml)
	AT = 8	AT = 22.9 ± 2.5								AT_post_ = 29.9 (ng/ml)
Erickson et al. (2011)	CG = 60	CG = 65.5 ± 5.44	Health	NA	Weekly/3 times	NA	moderate intensity aerobic exercise	6 months	Regular stretching.	AT_pre_ = 21.32 (pg/ml)
	AT = 60	AT = 67.6 ± 5.81								AT_post_ = 23.77 (pg/ml)
Jeon and Ha (2017)	CG = 10	CG = 15.05 ± 0.41	Health	NA	Weekly/4 times	70 % VO_2max_	200 kcal consumption per aerobic exercise	12 weeks	Stretching exercises	AT_pre_ = 25.24 (ng/ml)
	AT = 10	AT = 15.15 ± 0.33								AT_post_ = 30.09 (ng/ml)
Byun and Kang (2016)	CG = 11	CG = 70.46 ± 2.85	Health	50 min	Weekly/4 times	9-14-point on the Borg Scale (RPEs)	13 exercise movements to improve upper and lower body strength and aerobic endurance	12 weeks	No intervention	AT+RT_pre_ = 19.07 (ng/ml)
	AT+RT = 13	AT+RT = 70.45 ± 4.18								AT+RT_post_ = 20.1 (ng/ml)
Seifert et al. (2010)	CG = 5	CG = 31 ± 7	Health	45 min	Weekly/3 times	70% HR or 65% VO_2max_	Included mainly cycling, but the subjects were also allowed to run, swim, or use a rowing	3 months	NA	AT_pre_ = 2.5 (ng/ml)
	AT = 7	AT = 29 ± 6								AT_post_ = 5.5 (ng/ml)
Ruiz et al. (2015)	CG = 20	CG = 92.1 ± 2.3	Health	45 min	Weekly/3 times	30–70%(1-RM)	Mainly with the machine on the lower limb muscle strength training, also included 1 set of 8-10 repetitions of biceps curls, arm extensions, arm side lifts, shoulder elevations, seated bench press and leg calf rise, using dumbbells (1-3 kg per exercise) or low-to-medium resistance bands.	8 weeks	Stretching	RT_pre_ = 14.02 (ng/ml)
	RT = 20	RT = 92.3 ± 2.3								RT_post_ = 12.00 (ng/ml)
Kim et al. (2015)	CG = 32	CG = 81.8 ± 2.8	Health	60 min	Weekly/3 times	NA	Lower body exercises consisted of leg extensions, hip flexions, and more. Upper body exercises included double arm pull downs, bicep curls, and others balance and gait training	3 months	Placebo administration	CT_pre_ = 6.37 (ng/ml)
	CT = 33	CT = 80.3 ± 3.3								CT_post_ = 7.70 (ng/ml)
Fragala et al. (2014)	CG = 12	CG = 70.6 ± 6.1	Health	NA	Weekly/2 times	NA	Exercises included leg extensions, leg curls, seated rows, lat pull-downs, modified squats, modified split squats, modified stiff-legged dead-lifts, biceps curls, chest presses, shoulder presses, tricep extensions, abdominal exercises, and calf raises; 3 sets of 8–15 repetitions of each movement exercise	6 weeks	NA	RT_pre_ = 30.8 (ng/ml)
	RT = 13	RT = 70.6 ± 6.1								RT_post_ = 28.6 (ng/ml)
Maass et al. (2016)	CG = 19	CG = 68.4 ± 4.3	Health	40 min	Weekly/3 times	65% THR	Interval training on a stationary treadmill	3 months	Stretching training	AT_pre_ = 17.64 (ng/ml)
	AT = 21	AT = 68.4 ± 4.3								AT_post_ = 16.91 (ng/ml)
Hvid et al. (2017)	CG = 25	CG = 82.2 ± 4.5	Health	NA	Weekly/2 times	1–7 weekly: 70% RM, 8–12 weekly: 80% RM	The specific power training involved the following exercises: horizontal leg press, knee raises, plantar flexion, sitting Olympic lifts with dumbbells, lateral pull-down, lower back and abdominal exercises using elastic bands.	12 weeks	No intervention	RT_pre_ = 28.53 (ng/ml)
	RT = 22	RT = 82.7 ± 5.4								RT_post_ = 28.39 (ng/ml)
Matura et al. (2017)	CG = 24	>65	Health	30 min	Weekly/3 times	55–73% VO_2max_	Supervised bike riding training	12 weeks	No intervention	AT_pre_ = 3.718 (ng/ml)
	AT = 29									AT_post_ = 3.807 (ng/ml)
De Lima et al. (2022)	AT = 12	AT = 40.5 ± 5.63	Health	NA	Weekly/3 times	AT: 60–75% MVP; HIIT: 85−100% MVP	AT: running at a constant speed on a 300 m long track; HIIT: repeated 200 m sprints (10 × 20 m) interspersed with 1-min bouts of passive recovery.	8 weeks	NA	AT_pre_ = 1121.99 (pg/ml)
	HIIT = 13	HIIT = 39.46 ± 5.44								AT_post_ = 1852.41 (pg/ml)
										HIIT_pre_ = 1204.82 (pg/ml)
										HIIT_post_ = 2010.54 (pg/ml)
Rezola-Pardo et al. (2020)	CG = 35	≥70	Health	60 min	Weekly/2 times	40–70% (1-RM)	Personalized upper and lower body exercises, balance training and final stretching	3 months	Walking	CT_pre_ = 25.88 (ng/ml)
	CT = 32									CT_post_ = 26.94 (ng/ml)
Yin et al. (2022)	CG = 15	CG = 74.77 ± 6.04	Health	40–70 min	Weekly/3 times	45–75% THR	Perform moderate-intensity comprehensive training, exercise content includes jogging, stretching, resistance training of large muscle groups, balance and coordination training.	20 weeks	Maintain past habits	CT_pre_ = 7.61 (ng/ml)
	CT = 15	CT = 73.83 ± 7.51								CT_post_ = 14.45 (ng/ml)
Wens et al. (2016)	CG = 7	CG = 44 ± 5.29	MS	45–75 min	2 weeks/5 times	12-14-point on the Borg Scale (RPEs)	The first part of the training session is cycling and treadmill running, exercise intensity gradually increased, the second part is strength training (leg press, leg curl, leg extension, vertical traction, arm curl and chest press), the number of intervention groups and the number of times gradually increased.	24 weeks	Sedentary control	AT+RT_pre_ = 11092 (ng/ml)
	AT+RT = 15	AT+RT = 42 ± 11.62								AT+RT_post_ = 12020 (ng/ml)
Deus et al. (2021)	CG = 76	CG = 66.33 ± 3.88	HP	60 min	Weekly/3 times	NA	Each exercise needs to complete 7 movements(unilateral chest press _‘_squat _‘_ bilateral knee extension _‘_ hip thrust _‘_biceps curl _‘_ unilateral elbow extension _‘_ seated calf raise), supervised by a professional fitness trainer.	6 months	Routine care	RT_pre_ = 11.66 (ng/ml)
	RT = 81	RT = 67.27 ± 3.24								RT_post_ = 19.60 (ng/ml)
Imboden et al. (2021)	CG = 20	CG = 38.3 ± 13.4	Depression	40–50 min	Weekly/3 times	60–75% HR_max_	Indoor aerobic cycling under the supervision of a trainer.	6 weeks	Stretching exercise.	AT_pre_ = 23.0 (ng/ml)
	AT = 22	AT = 41.3 ± 9.2								AT_post_ = 30.3 (ng/ml)
Nascimento et al. (2015)	CG = 21	CG = 67.45 ± 4.9	CI	60 min	Weekly/4 times	70–80% HR_max_	Including RT for large muscle groups, AT, and balance training at an exercise.	16 weeks	Routine care	CT_pre_ = 2.44 (pg/dl)
	CT = 24	CT = 67.6 ± 6.1								CT_post_ = 3.07 (pg/dl)
Kohanpour et al. (2017)	CG = 10	CG = 67.85 ± 3.89	CI	40 min	Weekly/3 times	75–85% HR_max_	Aerobic run	12 weeks	No intervention	AT_pre_ = 110.25 (pg/ml)
	AT = 10	AT = 67.85 ± 3.89								AT_post_ = 192.84 (pg/ml)
Zimmer et al. (2018)	CG = 30	CG = 48 ± 12.1	MS	20 min	Weekly/3 times	85–90% HR_max_	High-intensity intervals of cycling	3 weeks	Routine care	HIIT_pre_ = 20.965 (ng/ml)
	HIIT = 27	HIIT = 51 ± 9.9								HIIT_post_ = 24.663 (ng/ml)
Kerling et al. (2017)	CG = 20	CG = 40.9 ± 11.9	Depression	45 min	Weekly/3 times	50% of the maximum workload	Cycling 25 min, remaining 20 min according to personal preference independent choice of exercise.	6 weeks	Conventional treatment	CT_pre_ = 415.20 (pg/ml)
	CT = 22	CT = 44.2 ± 8.5								CT_post_ = 472.50 (pg/ml)
Ozkul et al. (2018)	CG = 18	CG = 34 ± 8.7	MS	90 min	Weekly/4 times	60–80% HRR	30 min of aerobic exercise and 60 min of Pilates training exercise	8 weeks	Balance training	CT_pre_ = 696.23 (pg/ml)
	CT = 18	CT = 34.5 ± 12.78								CT_post_ = 891.15 (pg/ml)
Abbaspoor et al. (2020)	CG = 8	CG = 36.75 ± 6.8	MS	15–20 min	Weekly/3 times	55–70% HRR	Including static/dynamic stretching, aerobic running, RT, and balance training	8 weeks	No intervention	CT_pre_ = 1.96 (ng/ml)
	CT = 8	CT = 33.5 ± 6.37								CT_post_ = 1.79 (ng/ml)
Krogh et al. (2014)	CG = 38	CG = 43.8 ± 12.2	Depression	45 min	Weekly/3 times	80% HRR	Participants exercised on stationary bikes at approximately 80% of their maximal heart rate	3 months	Stretching exercises	AT_pre_ = 25.347 (ng/ml)
	AT = 41	AT = 38.9 ± 11.7								AT_post_ = 26.006 (ng/ml)
Elham and Masoud (2018)	CG = 12	CG = 31.41 ± 8.89	MS	30−40 min	Weekly/3 times	NA	The main exercises included Hundred, Roll-Up, Roll-Down, and Single Leg Circle movements, Increase the number of repetitions after the second month (which started with three to four repetations and gradually increased reached up to 10)	8 weeks	Maintain the previous habits	CT_pre_ = 10.68 (ng/ml)
	CT = 12	CT = 34.46 ± 7.29								CT_post_ = 11.55 (ng/ml)
Szymura et al. (2020)	CG = 13	CG = 65.23 ± 7.4	PD	30–60 min	Weekly/3 times	NA	Balance training with moderate-intensity exercise	12 weeks	NA	AT_pre_ = 21.19 (ng/ml)
	AT = 16	AT = 66 ± 2.59								AT_post_ = 30.37 (ng/ml)
Schulz et al. (2004)	CG = 12	CG = 39 ± 9	MS	30 min	Weekly/2 times	60% VO_2max_	Indoor cycling training	8 weeks	NA	AT_pre_ = 4353 (pg/ml)
	AT = 13	AT = 40 ± 11								AT_post_ = 5930 (pg/ml)
Liu et al. (2020)	AT = 31	AT = 84.68 ± 6.74	CI	30 min	Weekly /5 times	RT: 40–50%(1–RM); AT: 5–6 on a scale of perceived force	RT: exercise large muscle groups with the help of equipment; AT: indoor cycling training	4 weeks	NA	AT_pre_ = 19.25 (ng/ml)
	RT = 30	RT = 86.77 ± 6.99								AT_post_ = 21.20 (ng/ml)
										RT_pre_ = 23.46 (ng/ml)
										RT_post_ = 25.41 (ng/ml)
Hsu et al. (2021)	HIIT = 10	HIIT = 58.5 ± 20.06	SP	30–45 min	Weekly/3 times	HIIT: 80% VO_2max_	HIIT: Indoor cycling training	12 weeks	NA	HIIT_pre_ = 6.06 (ng/ml)
	AT = 13	AT = 53.1 ± 15.91				AT: NA	AT: moderate-intensity continuous training			HIIT_post_ = 7.91 (ng/ml)
										AT_pre_ = 7.3 (ng/ml)
										AT_post_ = 5.88 (ng/ml)
Frazzitta et al. (2014)	CG = 10	CG = 65 ± 4	PD	90 min	Weekly/5 times	NA	The main exercises include muscle stretching, balance and gait exercises, treadmill training	4 weeks	NA	CT_pre_ = 21.64 (ng/ml)
	CT = 14	CT = 67 ± 5								CT_post_ = 24.77 (ng/ml)
Briken et al. (2016)	CG = 10	CG = 50.4 ± 7.6	MS	20 min	Weekly/3 times	NA	Bicycle riding training, with stepwise progression in intensity and duration over a time of 9 weeks.	9 weeks	NA	AT_pre_ = 5.11 (ng/ml)
	AT = 32	AT = 49.9 ± 7.5								AT_post_ = 5.75 (ng/ml)
Damirchi et al. (2014)	CG = 10	CG = 55.37 ± 3.45	MD	60 min	Weekly/3 times	60% VO_2max_	Supervised running and aerobic exercise	12 weeks	Remain sedentary	AT_pre_ = 1112.91 (pg/ml)
	AT = 11	AT = 54.12 ± 2.77								AT_post_ = 1033.85 (pg/ml)
Yin et al. (2022)	CG = 14	CG = 70.73 ± 5.15	CI	40–70 min	Weekly/3 times	45–75% THR	Perform moderate-intensity comprehensive training, exercise content includes jogging, stretching, resistance training of large muscle groups, balance and coordination training.	20 weeks	Maintain past habits	CT_pre_ = 5.17 (ng/ml)
	CT = 12	CT = 69.08 ± 4.68								CT_post_ = 14.46 (ng/ml)

### Methodological quality assessment

**Figure 2:**
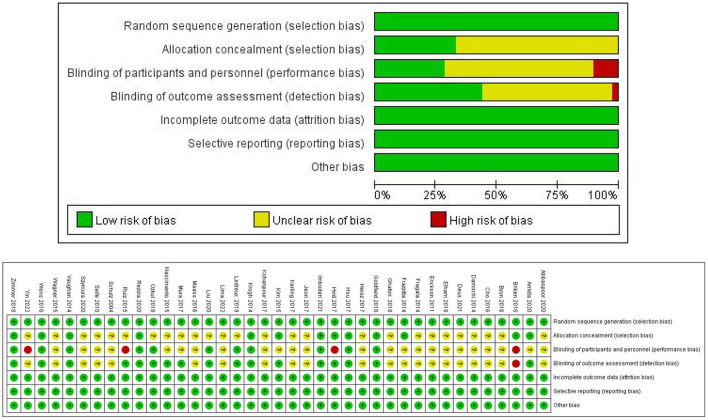
Risk of bias assessment diagram.

Of the 39 RCTs included, 16 trials reported a random sequence (random number table, random envelope, and computer), while the remaining 23 did not specify the random assignment method; 13 trials reported the random assignment concealment method: 10 trials were double-blinded, six trials were single-blinded, and one trial did not implement the blinding procedure. In addition, the remaining 22 trials did not report the blinding method. All trials reported the primary outcome indicators completely, with no selective reporting. Furthermore, all trials did not report other biases. The Cochrane Risk of Bias Assessment Tool was used to conduct a methodological assessment of the included trials, and the detailed results are shown in Figure 2.

### Network meta-analysis

#### Reticulated evidence map for the NMA

**Figure 3:**
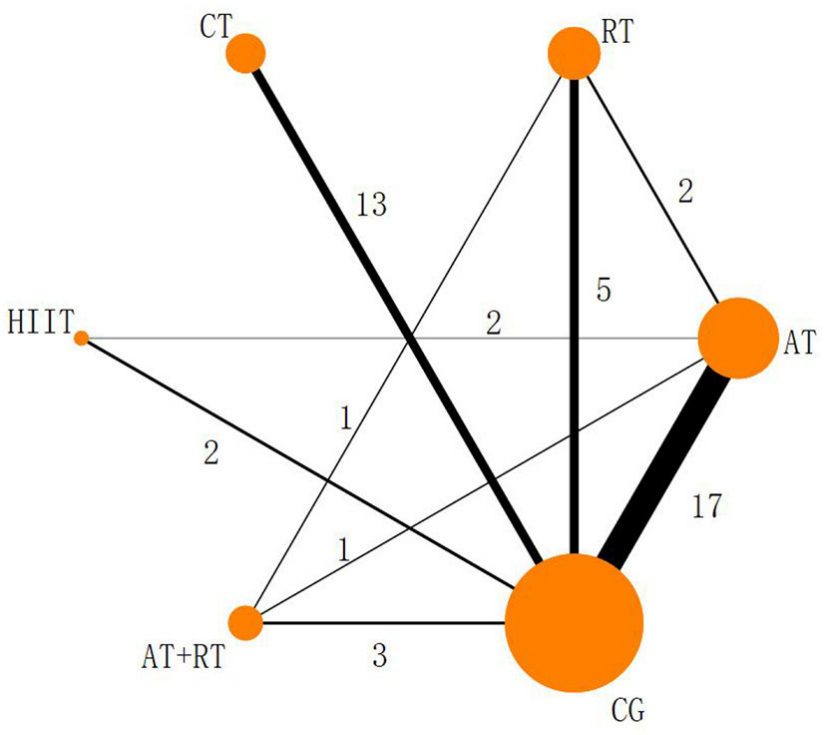
Reticulated evidence map of the effect of different exercise modalities on serum BDNF levels. Each node (solid circle) represents only one type of rehabilitation treatment. The size of the nodes is proportional to the number of subjects involved in the particular treatment intervention (i.e., sample size). The solid line connects the treatment with a direct comparison whose thickness is proportional to the number of trials.

The five physical activities constructed a CG-centered mesh evidence map (Figure 3) with a total of six intervention nodes and two closed loops: CG–AT–RT–AT+RT and CG–AT–HIIT (Figure 3). The nodal analysis suggested that the *P*-values were all greater than 0.05 (Table 4), indicating good agreement between direct and indirect comparisons among the 39 included trials. Therefore, the result data for the consistency test was used in this study.

**Table 4:** The consistency of nodal analysis direct and indirect comparisons.

**Name**	OR(95% CI)/SMD(95% CI)*			***P*** **-value**
	**Direct effect**	**Indirect effect**	**Overall**	
AT,RT	−0.57 (−7.37, 6.43)	−0.56 (−4.84, 3.75)	−0.66 (−4.11, 2.79)	0.98
AT,HIIT	−1.25 (−2.87, 0.28)	0.05 (−3.98, 4.01)	−1.11 (−2.61, 0.32)	0.55
AT,AT+RT	0.99 (−2.94, 4.88)	−1.01 (−6.23, 4.24)	0.32 (−2.79, 3.39)	0.53
AT,CG	2.57 (−2.11, 7.25)	1.81 (−2.01, 5.56)	2.00 (−0.95, 4.86)	0.80
RT,AT+RT	1.24 (−5.81, 8.22)	3.27 (−1.26, 7.77)	2.65 (−1.43, 6.69)	0.61
RT,CG	0.25 (−4.89, 5.39)	2.29 (−1.86, 6.54)	1.45 (−1.79, 4.56)	0.53
HIIT,CG	2.54 (−0.66, 5.53)	5.37 (−1.31, 11.92)	3.10 (0.38, 5.81)^*^	0.43

* *Effectiveness rate was OR (95% CI) and the remaining indicators were SMD (95% CI).*

#### Results of NMA

Total BDNF scores were reported in 39 trials. We compared the intervention outcomes between various intervention groups and the control group using MD (as the effect size) and 95% CI. Subjects in the RT [MD = 3.11 (0.33, 5.76), *P* < 0.05] and CT groups [MD = 1.68 (0.13, 3.29), *P* < 0.05] had significantly better outcomes than those in the control group. In addition, all exercise treatments significantly improved BDNF. However, other two-by-two comparisons between intervention groups did not show statistically significant differences (*P* > 0.05, **Table 6**).

#### NMA-derived probability ranking

**Figure 4:**
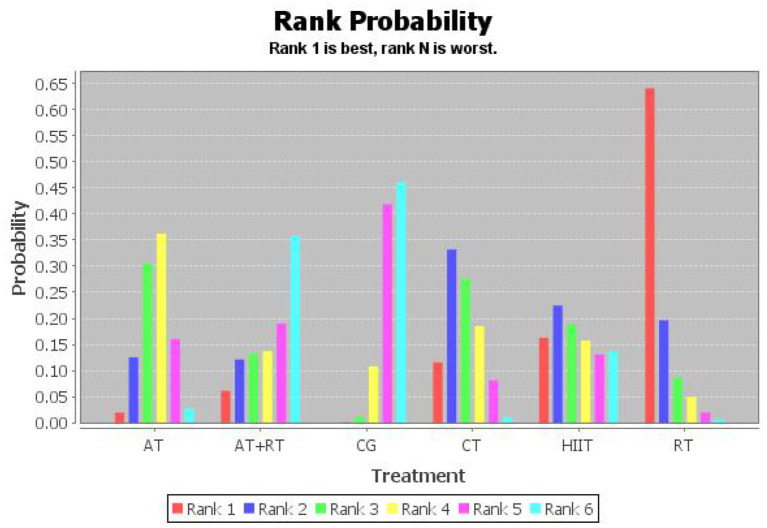
Probability ranking chart of the effect of five types of exercise on BDNF.

To investigate which exercise strategy had the greatest effect on serum BDNF levels in healthy and non-healthy populations, the intervention effects of the five non-pharmacological treatments were subjected to probability ranking. Statistical evidence revealed that the effect of exercise on BDNF level improvement in healthy and non-healthy people was ranked as follows: RT > HIIT > CT > AT+RT > AT > CG (Figure 4 and Tables 5, 6).

**Table 5:** Results of NMA.

**BDNF Score**					
RT		**Certainty of evidence**			
1.67 (−2.48, 5.61)	HIIT	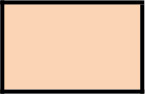 High	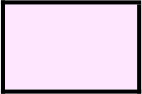 Moderate	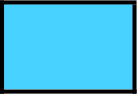 Low	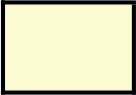 Very low
1.43 (−1.82, 4.53)	−0.26 (−3.77, 3.28)	CT			
3.11 (0.33, 5.76)*	1.43 (−1.70, 4.66)	1.68 (0.13, 3.29)*	CG		
2.63 (−1.51, 6.65)	0.97 (−3.56, 5.49)	1.22 (−2.44, 4.82)	−0.47 (−3.76, 2.71)	AT+RT	
1.98 (−0.94, 4.80)	0.32 (−2.74, 3.38)	0.55 (−1.60, 2.68)	−1.12 (−2.58, 0.34)	−0.65 (−4.15, 2.85)	AT

The outcome analysis-derived ranking table shows the percentage change in BDNF level relative to baseline data. The ranking table shows only the relative effects of each exercise on BDNF levels (interventions in the columns vs. those in the rows). In addition, relative effects are expressed as the standardized mean difference in percent change in BDNF and 95% Cl. *indicates a statistically significant difference. The color of each table cell indicates the recommended evidence for assessment and development and the level of assessment determined. All tables list treatments in alphabetical order.

**Table 6:** Probability ranking results of different outcome indicators for each intervention.

**Interventions**	**AT**	**AT+RT**	**CG**	**CT**	**HIIT**	**RT**
BDNF Score	0.02	0.06	0.00	0.11	0.16	0.66

#### Publication bias

**Figure 5:**
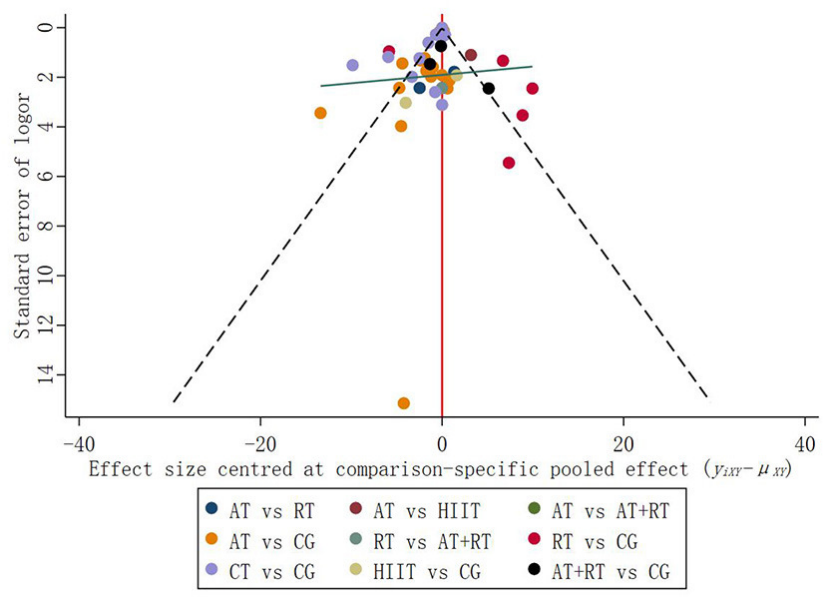
Funnel plot of the effects of five exercise interventions on BDNF levels.

In addition to direct comparisons, some of the trials included in NMA involved indirect comparisons and subjects that had not yet undergone a comparison. Therefore, relevant adjustments were required for control groups with different publication biases. In the funnel plot, studies with small sample sizes have low precision and are scattered all over the bottom of the funnel plot; studies with large sample sizes have high precision and are concentrated at the top of the funnel plot (Galbraith, 1988). The results suggested that the distribution of trials on both sides of the vertical line at *X* = 0 is symmetrical, and the balance line exhibits a slight inclination pattern (low on the left and high on the right) (Figure 5). The scatter of interventions was concentrated at the top of the funnel plot, with only one point at the bottom of the funnel plot, suggesting a low likelihood of publication bias in the included studies.

## Discussion

According to the retrieved information, this is the first RCT Bayesian Network Meta-analysis using a multiple comparison approach to study the effect of physical activity interventions on BDNF levels in healthy vs. non-healthy populations. Compared to traditional Meta-analysis (Marinus et al., 2019; Ruiz-González et al., 2021), the current study gives a more definitive answer—resistance training is the best exercise to increase peripheral BDNF in both older and younger individuals (healthy or diseased individuals) (see Supplementary material 2 for statistical results). Statistical evidence suggests that exercise is effective in improving peripheral BDNF levels in adolescents and older adults (healthy and diseased individuals) for all types of exercise, and our findings are consistent with those of the Ruiz-González et al. (2021) study. The current study explains the controversy that exists regarding the effects of different exercise interventions and provides more convincing evidence that physical activity promotes brain health.

**Figure 6:**
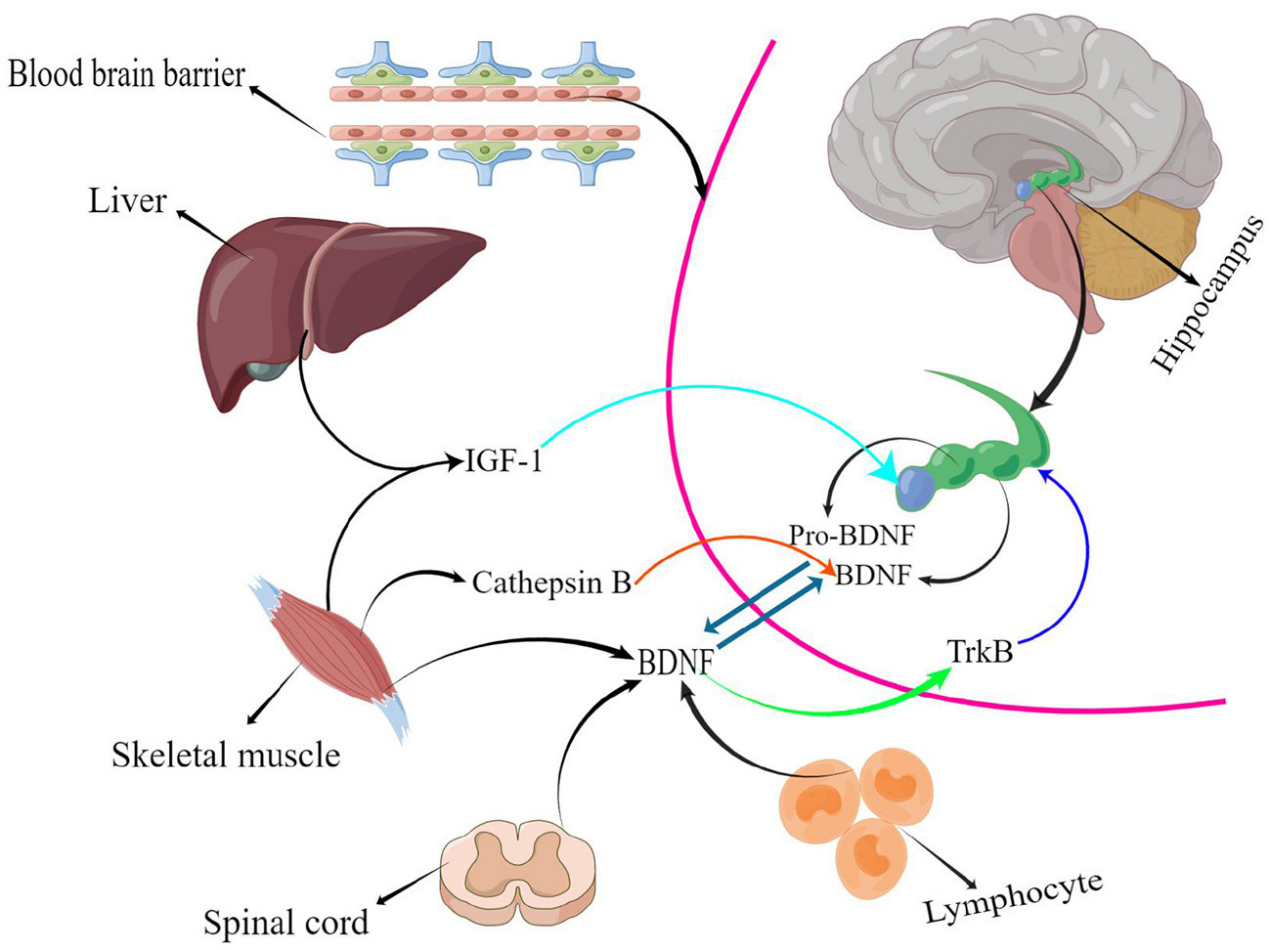
Exercise mediated peripheral cytokines(By Figdraw).

The hippocampus, the body's memory switch, regulates storage conversion and long-term memory orientation. Magnetic resonance imaging (MRI) suggests that the hippocampus in the brain decreases in size with age or the progression of depression and other NSDs in adulthood (Driscoll et al., 2003; Von Bohlen und Halbach, 2010), resulting in a reduction in learning, memory, and emotional control. In addition, the reduction in hippocampal volume causes a range of adverse events, such as a decrease in the number of brain neurons (Issa et al., 1990) and synaptic connections (Geinisman et al., 1995), as well as a decrease in BDNF levels (Lommatzsch et al., 2005). Studies in adolescents have confirmed that children with intellectual deficits and learning disabilities have a positive correlation between their learning ability and BDNF levels; BDNF mediates neurogenesis, and exercise improves BDNF levels to improve learning ability (Ghafori et al., 2018), which is one of the reasons for improved executive and cognitive abilities in the elderly. Numerous animal studies have indicated that exercise increases serum BDNF and IGF-1 levels *in vivo*, and the structural, functional, and cognitive effects of these factors on the hippocampal region are beneficial (Bechara and Kelly, 2013; Cetinkaya et al., 2013). Active athletes had higher BDNF levels than sedentary individuals (Correia et al., 2011). This confirms that sport is medicine. There is a long-standing consensus on the efficacy of exercise to enhance cardiorespiratory fitness, promote brain health, and prevent and mitigate a range of neurodegenerative diseases, such as Alzheimer's disease and Parkinson's disease (Bangsbo et al., 2019; Mintzer et al., 2019). However, determining which exercise has the best effect on people's BDNF levels is the focus of this study. Statistical results suggested that RT has the best effect on BDNF levels (Figure 4). RT has great potential to increase BDNF levels; in addition, BDNF released during skeletal muscle contraction flows to the brain and activates multiple signaling pathways (Deus et al., 2021). Cathepsin B (CTSB) levels are elevated in the gastrocnemius muscle and plasma following exercise stimulation; this type of muscle-secreted factor can cross the BBB to modulate BDNF concentrations, promoting brain plasticity and ultimately improving cognitive and memory function (Moon et al., 2016). Furthermore, CTSB has neuroprotective effects, and brain atrophy was present in mice whose CTSB genes were knocked out (Felbor et al., 2002). Therefore, exercise-induced increase in CTSB levels is a crucial protective factor. Several studies have also found a correlation between a decrease in muscle mass or an increase in body fat percentage and memory loss and cognitive decline (Beeri et al., 2021; Anand et al., 2022; De Las Heras et al., 2022). RT prevents muscle atrophy and increases the expression of muscle secretory factors, exerting a better effect on brain health than other forms of physical activity. In peripheral cells and organs BDNF is mainly expressed in muscle tissue (Huang et al., 2014), it is also found in the spinal cord and lymphocyte tissues (Gómez-Pinilla et al., 2001; Casoli et al., 2014). BDNF binds specifically to tropomyosin receptor kinase B (TrkB) to regulate the development, survival, and differentiation of brain neurons and stimulates neurogenesis in the hippocampal region (Gray et al., 2006; Araya et al., 2013; Figure 6). In summary, bones and muscles are important tissues for peripheral BDNF secretion, and resistance training causes more intense stress in bones and muscles, which stimulates BDNF expression in peripheral tissues, transport to the brain *via* blood circulation, and effects on the brain after crossing the BBB.

In addition to BDNF, IGF-1 plays a vital role in maintaining brain health by actively regulating brain function through the BBB (Lewitt and Boyd, 2019). Changes in IGF-1 levels are positively correlated with changes in hippocampal volume (Hvid et al., 2017), which provides nutritional support to neurons and reverses brain aging (Sonntag et al., 1999). Furthermore, beneficial secreted factors, such as IGF-1, are widely expressed during human growth and development but their levels are decreased in adulthood. IGF-1 is predominantly expressed in the liver but is also found in other tissues such as the bone and skeletal muscle. Exercise stimulation increases IGF-1 secretion (Ye et al., 2020). A systematic review by Ye et al. (2020) reported a significant increase in serum IGF-1 levels in subjects older than 60 years who engaged in RT. Similarly, a 12-year longitudinal study suggested that loss of skeletal muscle mass with age was associated with a decrease in serum IGF-1 levels (Frontera et al., 2000). Skeletal muscle is the primary tissue source for systemic IGF-1 production during high-intensity physical activity (Ye et al., 2020). Therefore, physical activity is extremely essential in maintaining brain health.

Research on the effects of BDNF on brain function has greatly benefited from the identification of single nucleotide polymorphisms (SNPs) in the gene encoding human BDNF, which converts valine to methionine at codon 66 (Val66Met), and from *in vitro* incubation experiments demonstrating that altered amino acid polymorphisms at codon 66 in the BDNF gene affect the intracellular distribution, packaging and release of BDNF protein *in vitro*. packaging and release *in vitro* (Egan et al., 2003; Lu et al., 2013). In addition, the BDNF Val66Met polymorphism may affect plasma BDNF concentrations and TrkB receptor activity in peripheral tissues, and may impair the regulated secretion and intracellular transport of BDNF (Lemos Jr et al., 2016; Trombetta et al., 2020). It has been shown that individuals carrying the BDNFMet allele have a reduced exercise gain effect, which seems to explain the consistent lack of significant BDNF changes after exercise in some subjects, regardless of their health (Lu et al., 2013; Lemos Jr et al., 2016). Notably, when participants possessed high levels of physical activity, their memory and cognitive performance remained consistently high whether they carried the BDNFMet allele or the BDNFVal allele. It is not difficult to find that maintaining good exercise habits can offset the potential cognitive disadvantage caused by the BDNF Val66Met polymorphism.

In addition to RT, HIIT, CT, AT+RT, and AT also have beneficial effects on other body functions and peripheral blood BDNF levels. Although some studies confirmed that exercise has no significant effect on BDNF levels, there is a lack of follow-up studies to demonstrate whether there is a decrease in BDNF levels after cessation of exercise. The current study showed that all five interventions achieved effective outcomes, suggesting that all types of exercise are beneficial. However, we observed that the effect of exercise on BDNF levels in the healthy population was not significant, which may be attributed to the consistently higher levels of serum BDNF in the healthy population. The increase in BDNF in healthy individuals may have plateaued because physical activity cannot drive an unrestricted increase in BDNF, demonstrating that regular physical activity maintains function-specific homeostasis in the body (Liu et al., 2014). BDNF levels were negatively correlated with aerobic capacity in healthy populations (Rezola-Pardo et al., 2020) and higher levels of fitness prevented a decrease in hippocampal volume (Erickson et al., 2011), which plausibly explains the non-significant effect of exercise on BDNF levels in healthy populations.Neither physical exercise nor other interventions can repair what is not damaged. Finally, we used the quantitative difference(QD) statistical method proposed by research group of Liu et al. (2017); Sun et al. (2019) to reanalyze the geometric mean (Waldinger et al., 2008; Moser et al., 2020) (calculated from the baseline mean and endpoint means) of the intervention results of the 39 papers included in this network meta-analysis. The study interventions of Yin et al. (2022) and Seifert et al. (2010) were found to have a significant effect on BDNF levels in the chronically inactive population, with QD values greater than α at the level of cells, molecules or central nervous systems. Suggesting that CT and AT may have more significant activation of specific signal transduction pathways in the non-exercise habit group, thereby inducing BDNF expression in skeletal muscle cells. It is important to note that the reasons for its QD value failed to exceed α at the level of cells, molecules or central nervous systems from reanalysis of the other 37 studies may be related to the rigor of the study design, the failure to investigate the previous exercise habits of the intervention population, or the recruitment of a population with active exercise behavior prior to the intervention, which will be verified by our group in a follow-up study. Therefore, exercise may be a specific factor to prevent the decline of BDNF level or keep it at an optimal level. Furthermore, exercise positively regulates BDNF and IGF-1 levels, which is beneficial for neuronal growth, development, and survival. Consequently, it acts as a crucial protective factor in mediating exercise-induced improvements in cognition and reducing depression to prevent the continued progression of other neurodegenerative diseases.

The mechanism by which exercise regulates BDNF and IGF-1 levels to improve brain health is unknown. We speculate, as active stimulation, exercise mediates stress in skeletal muscle or other tissues and organs in response to injury or inflammation, thereby initiating the repair process. BDNF and IGF-1 cross the BBB for the repair of injured and aging brain tissues.

### Research prospects

Maintaining optimal levels of BDNF is critical for physical and brain health. Modern advances in biology have made it easier to extract or synthesize BDNF from other animal tissues. However, the special structure of the BBB prevents harmful substances from entering the CNS, and also reduces the penetration of exogenous drugs through the BBB, and affects the treatment of brain diseases. This is attributed to the lack of curative drugs for other degenerative neurological diseases such as depression and Alzheimer's disease. Endogenous BDNF produced by exercise, on the other hand, can bind specifically to TrkB, which crosses the BBB for the repair of injured and aging brain tissues. In future studies, we will focus on the effect of exercise on BBB permeability and the ameliorative effect of exogenous BDNF on degenerative neurological disorders when used after exercise. In the end, more evidence of high-intensity interval training interventions is desired to determine if resistance training is the optimal intervention.

### Limitations

This study also has several limitations. In the quantitative consolidation, the means and standard deviations presented partially in the form of graphs were extracted through GetData, which may have led to some errors. Additionally, only Chinese and English databases were searched. Finally, small sample studies were included.

## Conclusion

Bayesian Network Meta-Analysis evidence suggests that better outcomes were observed in all five intervention groups than that in the conventional control group. The effect of exercise on BDNF level improvement in healthy and non-healthy populations was ranked as follows: RT > HIIT > CT > AT+RT > AT > CG. Exercise has a polypill effect, consequently activating endogenous disease resistance mechanisms. Physical activity as a positive non-pharmacological stimulus enables organs or tissues to initiate the release of endogenous drugs to fight internal diseases of the organism due to aging. RT at moderate intensity is recommended for children and older adults in the case of exercise tolerance, which is more effective in maintaining or increasing BDNF levels for brain health compared to other exercise types. In summary, exercise is a simple and effective way to maintain brain function and promote brain remodeling. Finally, exercise prescriptions should take into account the exercise preferences of the target population to avoid noncompliance.

## Data availability statement

The original contributions presented in the study are included in the article/Supplementary material, further inquiries can be directed to the corresponding author/s.

## Author contributions

TL and FM designed this study. BZ and ZW ran the search strategy, collected data, and assess the quality of studies. LZ and GH rechecked data. BZ performed analysis and wrote the manuscript. TL, CC, and JH rechecked. BL uses get date software to extract graphical data. ZW, TL, and FM edited. ZW lead into QD statistical method and geometric mean in this discussion. All listed authors reviewed and revised the manuscript.

## Funding

This work was supported by the National Key Research and Development Program of China (2017YFB0403800).

## Conflict of interest

Author FM was employed by Qinghai Institute of Sports Science Limited Company. The remaining authors declare that the research was conducted in the absence of any commercial or financial relationships that could be construed as a potential conflict of interest.

## Publisher's note

All claims expressed in this article are solely those of the authors and do not necessarily represent those of their affiliated organizations, or those of the publisher, the editors and the reviewers. Any product that may be evaluated in this article, or claim that may be made by its manufacturer, is not guaranteed or endorsed by the publisher.

## Abbreviations

CNS: central nervous system
BDNF: brain-derived neurotrophic factor
NGF: nerve growth factor
IGF-1: insulin-like growth factor
FGF-2: fibroblast growth factor 2
VEGF: vascular endothelial growth factor
BBB: blood-brain barrier
CSF: cerebrospinal fluid
NSD: nervous system disease
AT: aerobic training
RT: resistance training
HIIT: high-intensity interval training
CT: combined training
RCT: randomized controlled trial
NMA: network meta-analysis
PRISMA: preferred reporting items for systematic review and meta-analyses
MCMC: Bayesian Markov Chain Monte Carlo
PSRF: potential scaling reduction factor
ELISA: enzyme-linked immunosorbent assay
MRI: magnetic resonance imaging
CTSB: cathepsin B
TrkB: tropomyosin receptor kinase B
QD: quantitative difference
HRR: heart rate reserve
VO_2max_: maximum oxygen uptake
HR_max_: maximum heart rate
THR: target heart rate
MVP: maximum velocity performance
SNPs: single nucleotide polymorphisms.
